# Intrauterine administration of human chorionic gonadotropin does not improve pregnancy and life birth rates independently of blastocyst quality: a randomised prospective study

**DOI:** 10.1186/s12958-015-0069-1

**Published:** 2015-07-04

**Authors:** Barbara Wirleitner, Maximilian Schuff, Pierre Vanderzwalmen, Astrid Stecher, Jasmin Okhowat, Libor Hradecký, Tomáš Kohoutek, Milena Králícková, Dietmar Spitzer, Nicolas H. Zech

**Affiliations:** IVF Centers Prof. Zech, Römerstrasse 2, 6900 Bregenz, Austria; Centre Hospitalier Inter Régional Edith Cavell (CHIREC), Braine-l’Alleud, Bruxelles Belgium; IVF Centers Prof. Zech, B. Smetany 2, 30100 Pilsen, Czech Republic; Department of Histology and Embryology, Charles University in Prague – Faculty of Medicine in Pilsen, Karlovarská 48, 30166 Pilsen, Czech Republic; IVF Centers Prof. Zech, Innsbrucker Bundesstr. 35, 5020 Salzburg, Austria

**Keywords:** Intrauterine hCG supplementation, Implantation, Blastocyst transfer, Birth rate, IVF

## Abstract

**Background:**

Successful embryo implantation depends on a well-timed maternal-embryonic crosstalk. Human chorionic gonadotropin (hCG) secreted by the embryo is known to play a key role in this process and to trigger a complex signal transduction cascade allowing the apposition, attachment, and invasion of the embryo into the decidualized uterus. Production of hCG was reported to be dependent on blastocyst quality and several articles suggested that intrauterine hCG injection increases pregnancy and implantation rates in IVF patients. However, no study has as yet analysed birth rates as final outcome. Our objective was to determine whether clinical outcome after blastocyst transfer can be improved by intrauterine injection of hCG and whether this is dependent on blastocyst quality.

**Methods:**

A prospective randomised study was conducted in two settings. In cohort A, hCG application was performed two days before blastocyst transfer. In cohort B, the administration of hCG occurred just prior to embryo transfer on day 5. For both cohorts, patients were randomised to either intrauterine hCG application or to the control group that received culture medium. Clinical outcome was analysed according to blastocyst quality of transferred embryos.

**Results:**

The outcome of 182 IVF-cycles (cohort A) and 1004 IVF-cycles (cohort B) was analysed. All patients received a fresh autologous blastocyst transfer on day five. Primary outcomes were pregnancy rates (PR), clinical pregnancy rates (cPR), miscarriage rates (MR), and live birth rates (LBR). No improvement of clinical outcome after intrauterine hCG administration on day 3 (cohort A) or day 5 (cohort B) was found, independently of blastocyst quality transferred. The final outcome in cohort A: LBR after transfer of top blastocysts was 50.0 % with hCG and 53.3 % in the control group. With non-top blastocysts, LBR of 17.1 % (hCG) and 18.2 % (control) were observed (n.s.). In cohort B, LBR with top blastocysts was 53.3 % (hCG) and 48.4 % (control), with non-top blastocysts it came to 28.7 % (hCG) and 35.0 % (control). The differences between the groups were statistically not significant. Furthermore, we investigated a possible benefit of hCG administration in correlation with female age. In both age groups (<38 years and ≥ 38 years) we found similar LBR after treatment with hCG vs. medium. A LBR of 47.1 % vs. 48.7 % was obtained in the younger group and 26.6 % vs. 30.8 % in the older group.

**Conclusions:**

In contrast to previous studies indicating a substantial benefit from intrauterine hCG application in cleavage stage embryo transfers, in our study we could not find any evidence for improvement of clinical outcome in blastocyst transfer cycles, neither with top nor with non-top quality morphology.

## Background

Successful implantation of the embryo depends on a complex embryo-maternal crosstalk. In fact, human reproduction is characterized by a high prevalence of pre-implantation embryo loss of up to 75 % and a high frequency of clinical miscarriages, suggested to exceed a rate of 10 % of all clinical pregnancies [1, 2]. Aside from euploidy and potential of transferred embryo(s), the receptivity of the endometrium is most crucial for successful implantation. Despite extensive research, the embryo-maternal dialogue that orchestrates the implantation process is still not fully understood.

Human chorionic gonadotropin (hCG) is a major player in implantation, being involved in decidualization of the endometrial stromal cells, trophoblast invasion, proliferation of uterine natural killer (uNK) cells, immunological modulation at the maternal–foetal interface, stimulation of endometrial angiogenesis, and maintenance of progesterone secretion by the corpus luteum [3–6]. It has been assumed that hCG is responsible for a shift in gene expression in the uterine endometrium towards receptiveness for the implanting embryo [7].

It is well known that hCG is one of the earliest secreted molecules in human cleavage stage embryos and is later produced by the syncytiotrophoblast [8, 9]. The secretion of hCG has been reported to be associated with potential as well as with morphology of the embryo. This is consistent with the finding that embryos with poor morphological grading display reduced ability to ‘attract’ endometrial cells to prepare for implantation [10, 11]. In fact, a recent prospective study indicates a supportive effect of intrauterine hCG administration in transfers with poor quality blastocysts [12].

In the early implantation period, hCG was shown to inhibit IGFBP-1, a member of the insulin-like growth factor-binding protein family, that prevents the implantation process by binding to α5β1-integrins on the cell-surface of invading trophoblasts [13]. Further, hCG upregulates the leukaemia inhibitory factor (LIF), vascular endothelial growth factors (VEGFs) and matrix metalloproteinase-9 (MMP-9)- all factors known to be essential for embryo attachment, placentation, trophoblast invasion, and a multitude of other important processes in establishing a pregnancy [13]. Several other *in vitro* and *in vivo* studies in animal model systems but few in human subjects indicate that hCG changes the paracrine milieu of the endometrium [14–18].

Culture of embryos in *in vitro* fertilization (IVF) involves an absence of intrauterine hCG signalling during early embryo development (the first 3 to 5 days, depending on the day of transfer). It was hypothesized that this circumstance could contribute to the relatively low implantation rates in IVF, even after pre-implantation genetic screening (PGS). Intrauterine supplementation of hCG might circumvent this effect. Mansour and co-workers were the first to investigate the impact of intrauterine hCG injections on IVF-patients in a randomised trial. They found that application of 500 IU hCG prior to cleavage stage embryo transfer (ET) significantly improves pregnancy rates (PR) [19]. Similar results were reported in further studies on cleavage stage ETs [12, 20–22]. However, in blastocyst transfers, a beneficial effect of intrauterine hCG administration could only be confirmed for low-quality blastocysts [12].

The objective of this study was to investigate whether the reported beneficial effect of intrauterine hCG administration on clinical outcome after IVF treatment can be confirmed in blastocyst transfers, taking into account the embryo quality. A single administration of hCG two days before (cohort A) or shortly prior to ET was tested (cohort B). In a large study population, pregnancy rate (PR), clinical PR (cPR), and for the first time also miscarriage rate (MR) and live birth rate (LBR) were evaluated.

## Methods

### Study population and study design

This prospective observational study was approved by the ethics committee of the Charles University in Prague, EC-No. 355. All of the patients were counselled, and a signed informed consent was obtained from all participants. Our single-centre study included data on 182 IVF-cycles with hCG administration two days prior to transfer of a fresh blastocyst (cohort A) and 1004 cycles with hCG administration on the day of ET (cohort B). Only single embryo transfer (SET) or double embryo transfer (DET) were performed, according to the medical history of patients (e.g. caesarean section, risk for hyperstimulation syndrome) and according to the patient’s decision. The primary objective was to elucidate whether intrauterine hCG supplementation supports the implantation process in blastocyst transfers. A secondary goal was to find out whether hCG has detrimental effects such as an increased rate of biochemical pregnancies or abortions after detection of the foetal heartbeat. The reason for the application of hCG on either d3 (cohort A) or day 5 (cohort B) was that it has not yet been elucidated what moment of administration will better support endometrium receptivity in blastocyst transfers.

**Fig. 1:**
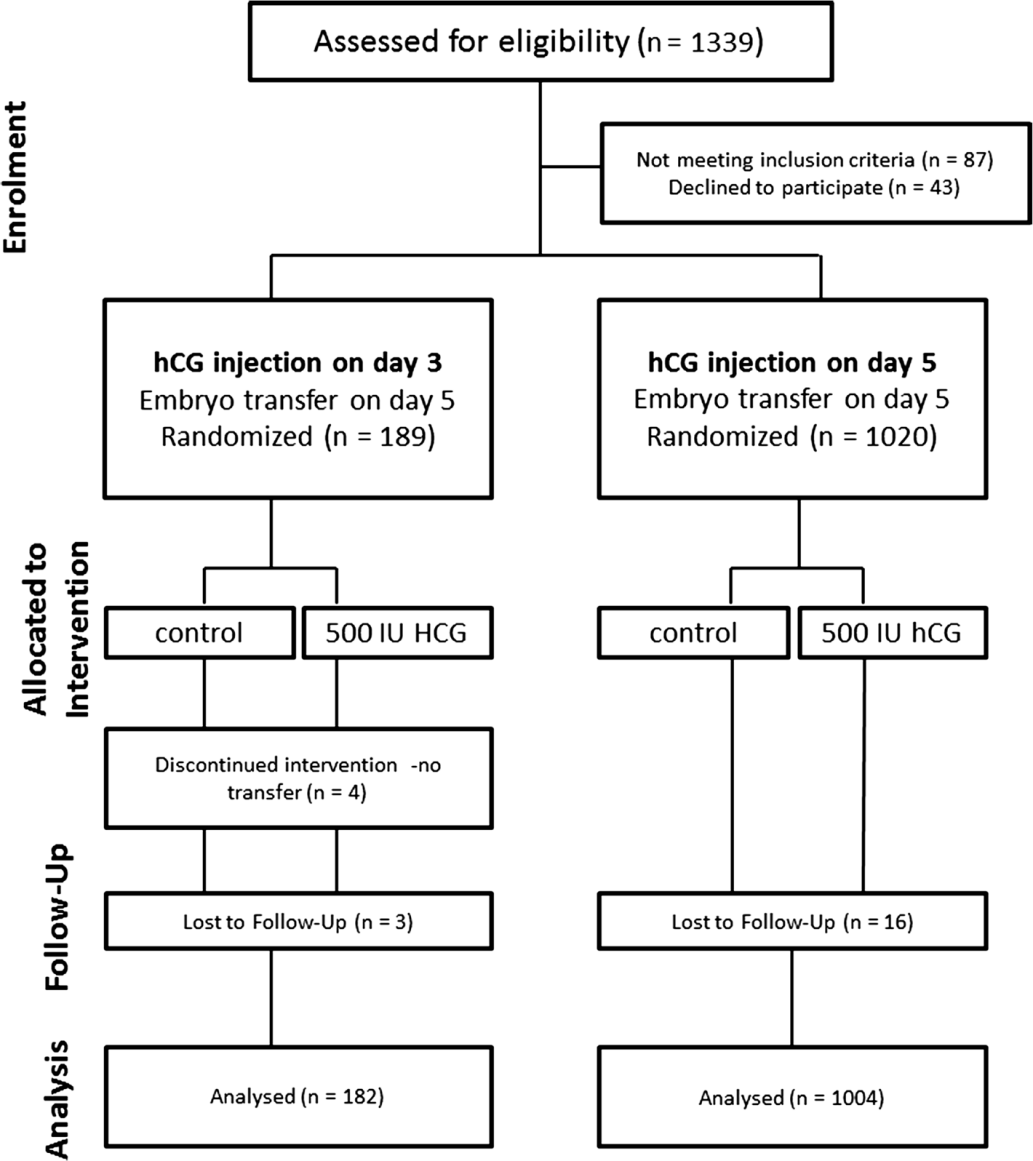
Flow chart diagram of the study. Out of 1339 patients who underwent an IVF therapy, 1252 were found to be eligible to participate. Forty-three patients declined to participate. Patients were randomized after oocyte retrieval in both cohorts. All patients underwent the same stimulation protocol (GnRHa long)

Patients were recruited between February 2013 and February 2014 and randomised to either administration of 500 IU hCG (Pregnyl, ORGANON, Netherlands) dissolved in 40 μl embryo culture medium G-2™ PLUS (Vitrolife, Sweden) or administration of the same amount of culture medium without hCG as control. Thereby, patients asking for implantation supporting medium transfer on day three were automatically allocated to cohort A. Counselling for implantation supporting medium transfer was done after one or two failed embryo transfers in the patient’s history. On the day of ovum pick-up (OPU), randomisation in both groups was done electronically with a random number generator using the Statistical Package for Social Sciences (SPSS) software version 17.0 for Windows (SPSS Inc., USA). Administration groups were patient blinded. Inclusion criteria for both groups were fresh autologous blastocyst transfer on day five and female age ≤ 43 years. Exclusion criteria were oocyte donation cycles and patients with reported recurrent implantation failure (≥3 negative IVF-cycles). A flow chart diagram demonstrates the study design, subject allocation, follow-up, and analysis (Fig. 1).

### Ovarian stimulation and oocyte retrieval

For ovarian stimulation, the GnRHa long protocol was applied [23]. The mean length of the stimulation period was 11.6 days, and the mean stimulation dose was 2824 IU follicle-stimulating hormone (FSH). No statistically significant differences in length and dose of stimulating hormones between the groups were found. The oocytes were retrieved 36 hours after intramuscular administration of 5.000 IU hCG (Pregnyl, ORGANON, Netherlands) (<0.2 % cycles) or 10.000 IU of hCG (>99.8 % cycles).

### Embryo culture

Based on the patients’ medical history, oocytes were fertilized either by standard insemination (IVF: 1.2 %), or intracytoplasmic morphologically selected injection (IMSI: 98.8 %) [24]. Embryo culture was performed in G-1™ PLUS and G-2™ PLUS media (Vitrolife, Sweden) in four-well dishes (Nunc A/S, Roskilde, Denmark). Medium was renewed on day 3. On day 5, blastocyst quality was evaluated, and the best or the best two blastocyst(s) in terms of morphology were selected for transfer. Morphological grading was performed based on the degree of blastocoele expansion and the appearance of the inner cell mass as well as the trophectoderm according to the classification of Gardner and colleagues [25]. Blastocysts with a degree of expansion of 2–5 and with A-grading for inner cell mass and trophectoderm or a combination of A- and B-grading were classified as top blastocysts. All other blastocysts were grouped together as non-top blastocysts.

### Cohort A: hCG administration on day 3

Administration of hCG in cohort A was done on day 3 after ovum pick-up (OPU) using an intrauterine insemination cannula (Gynetics, Lommel, Belgium). Therefore, 40 μl of either hCG (500 IU) dissolved in embryo culture medium or plain culture medium (control) were injected in the intrauterine space 0.5 cm below the fundus. Blastocyst transfer took place on day 5. Mean female age was 35.8 years (range 22–43 years), No statistically significant differences in patients’ characteristics [female age, body mass index (BMI), number of MII oocytes per OPU, type as well as cause of infertility, and percentage of IMSI and IVF] in the groups allocated to hCG or in the control group were found (Table 1).

**Table 1:** Patients’ characteristics of cohort A: hCG administration on day three

Characteristics	hCG (n = 89)	culture medium (n = 93)	*P*-Value
Female age (years) mean *± s.d.* (range)	36.1 *± 4.1* (22–43)	35.5 *± 5.0* (25–43)	0.365
BMI (kg/m^2^) mean *± s.d.*	23.0 *± 3.0*	23.6 *± 4.2*	0.383
No. MII oocytes/ OPU mean ± s.d.	9.2 *± 5.0*	9.0 *± 5.2*	0.796
Type of Infertility (%)			
-*Primary Infertility*	50 (56.2 %)	42 (45.2 %)	0.137
-*Secondary Infertility*	39 (43.8 %)	51 (54.8 %)	
Cause of Infertility (%)			
-*Female factor*	19 (21.4 %)	29 (31.3 %)	0.168
-*Male factor*	38 (42.3 %)	27 (29.0 %)	
-*Mixed*	25 (28.1 %)	32 (34.4 %)	
-*Idiopathic*	7 (7.9 %)	5 (5.4 %)	

Ranges or percentages respectively are given in brackets, MII oocytes: Metaphase II oocytes; OPU = ovum pick-up; s.d.: standard deviation

Baseline characteristics of IVF patients receiving either 40 μl of hCG (500 IU) dissolved in embryo culture medium or plain culture medium (control group) 2 days before transfer. Primary infertility was defined as the inability to become pregnant or the inability to carry a pregnancy to a live birth. Secondary infertility was defined as the inability to become pregnant or the inability to carry a pregnancy to a live birth following either a previous pregnancy or a previous ability to carry a pregnancy to a live birth

### Cohort B: hCG administration on day 5

Human chorionic gonadotropin was administered 3 minutes prior to blastocyst transfer on day 5 in cohort B. Therefore, either 40 μl hCG dissolved in embryo culture medium or 40 μl plain culture medium was applied to the surface of the endometrium using a Wallace embryo replacement catheter (Smiths Medical Inaternation, Kent, UK) 0.5 cm below the uterine fundus. Transfer of blastocysts was performed as described previously [26]. No statistically significant differences in patient characteristics between hCG and control groups were observed (Table 2).

**Table 2:** Patients’ characteristics in cohort B: hCG administration on day 5

Characteristics	hCG (n = 510)	culture medium (n = 494)	*P*-Value
Female age (years) mean *± s.d* (range)	*37.1 ± 4.0* (20–43)	36.*7 ± 4.3* (21–43)	0.102
BMI (kg/m^2^) mean *± s.d*	22.9 *± 4.4*	22.9 *± 4.3*	0.917
No. MII oocytes/ OPU mean *± s.d*	8.7 *± 5.0*	9.3 *± 5.6*	0.083
Type of Infertility (%)			
-*Primary Infertility*	249 (48.8 %)	236 (47.8 %)	0.752
-*Secondary Infertility*	261 (51.2 %)	258 (52.2 %)	
Cause of Infertility (%)			
-*Female factor*	77 (15.1 %)	63 (12.8 %)	0.451
-*Male factor*	220 (43.1 %)	229 (46.4 %)	
-*Mixed*	115 (22.5 %)	119 (24.1 %)	
-*Idiopathic*	98 (19.2 %)	83 (16.8 %)	

Ranges or percentages respectively are given in brackets, MII oocytes: Metaphase II oocytes; OPU = ovum pick-up; s.d.: standard deviation

Baseline characteristics of IVF patients receiving either 40 μl hCG dissolved in embryo culture medium or 40 μl plain culture (control) on day 5 after oocyte retrieval 3 minutes prior to transfer. Primary infertility was defined as the inability to become pregnant or the inability to carry a pregnancy to a live birth. Secondary infertility was defined as the inability to become pregnant or the inability to carry a pregnancy to a live birth following either a previous pregnancy or a previous ability to carry a pregnancy to a live birth

### Outcome measures

Fourteen days after blastocyst transfer, urinary ß-hCG was tested to determine the pregnancy rate (PR). Clinical pregnancy rate (cPR) was confirmed by at least one foetal heartbeat observed by ultrasound 8 to 12 weeks after transfer. The implantation rate (IR) was calculated by number of foetal heartbeats per transferred blastocyst. LBR is the number of deliveries per blastocyst transfer. Miscarriage was defined as pregnancy loss after detection of positive heartbeat by ultrasound in gestation week 8 to 12

### Statistical analysis

Primary study outcomes were IR, PR, cPR, and LBR. All rates were compared by a chi-square distribution and by calculating 95 % confidence intervals of the relative risk and risk difference. An alpha error of less than 0.05 was considered statistically significant. Power analysis was performed, revealing that 376 patients in each cohort are needed to detect a 10 % difference in birth rate with 80 % power and a single-tailed alpha of 0.05. Differences in means of numerical data were tested by one-way Anova using the F distribution. Statistical analysis was performed using the Statistical Package for Social Sciences (SPSS) software version 17.0 for Windows (SPSS Inc., USA).

## Results

### Cohort A: hCG administration on day 3

Groups were subdivided according to the blastocyst quality of transferred embryos. Ninety-seven women had transfers with top blastocysts. Forty-eight patients were randomised to the hCG group, whereas the control group of 49 patients had an uterine injection of embryo culture medium 2 days prior to transfer (Table 3). No statistically significant differences in female age or number of embryos transferred were found between the groups. In the follow-up, no difference in PR (60.4 % vs. 65.3 %, respectively), cPR (52.1 % vs. 57.1 %, respectively), LBR (50.0 % vs. 53.1 %, respectively), or MR (2.1 % vs. 4.1 %, respectively) was observed after administration of intrauterine hCG vs. medium.

**Table 3:** Clinical outcome in cohort A: hCG administration on day 3

	Transfer with top blastocysts			Transfer with non-top blastocysts		
Characteristics	hCG (n = 48)	culture medium (n = 49)	*P*-Value	hCG (n = 41)	culture medium (n = 44)	*P*-Value
Female age (years) mean *± s.d.* (range)	35.3 *± 4.2* (22–42)	34.9 *± 5.0* (25–42)	0.641	37.0 *± 3.9* (30–43)	36.1 *± 5.0* (25–43)	0.380
Endometrium height (mm) mean *± s.d.*	11.0 *± 2.6*	10.8 *± 2.4*	0.817	11.6 *± 2.2*	10.8 *± 1.9*	0.110
No. Embryos transferred Embryos / ET mean *± s.d.*	75 1.6 *± 0.5*	76 1.6 *± 0.5*	0.911	69 1.7 *± 0.5*	77 1.8 *± 0.5*	0.522
Proportion of difficult ETs	10.4 %	8.2 %	0.702	12.2 %	9.1 %	0.642
No. Pregnancies (PR)	29 (60.4 %)	32 (65.3 %)	0.618	13 (31.7 %)	13 (29.5 %)	0.829
No. Clinical pregnancies (cPR)	25 (52.1 %)	28 (57.1 %)	0.617	8 (19.5 %)	9 (20.5 %)	0.914
No. Implanted embryos (IR)	30 (40.0 %)	32 (42.1 %)	0.793	11 (15.9 %)	12 (15.6 %)	0.953
No. Births (LBR)	24 (50.0 %)	26 (53.1 %)	0.763	7 (17.1 %)	8 (18.2 %)	0.893
No. Babies born	27	30		9	9	
No. Miscarriages (MR)	1 (2.1 %)	2 (4.1 %)	0.570	1 (2.4 %)	1 (2.3 %)	0.960

Ranges or percentages respectively are given in brackets, s.d.: standard deviation

IVF characteristics and clinical outcome of cohort A. Miscarriage rate was defined as pregnancy loss after the detection of foetal heartbeat

In patients receiving transfers of non-top blastocysts, 41 women were allocated to the hCG injection prior to transfer and 44 women to injection with medium (control group). Female age, endometrium build-up, and number of transferred embryos were comparable in both groups (Table 3). No statistically significant differences in PR (31.7 % vs. 29.5 %, respectively), cPR (19.5 % vs. 20.5 %, respectively), LBR (17.1 % vs. 18.2 %, respectively), and MR (2.4 % vs. 2.3 %, respectively) were found after intrauterine hCG vs. medium injection.

The twin birth rate after transfer of top blastocysts was 12.5 % (hCG) vs. 15.4 % (control), with transfer of non-top blastocysts 28.6 % (hCG) vs. 12.5 % (control). The differences were not statistically significant.

### Cohort B: hCG administration on day 5

#### Impact of hCG application in relation to blastocyst quality

A total of 357 patients in this cohort received a transfer with top blastocyst(s), 169 of which were allocated to intrauterine hCG injection and 188 to injection with medium (Table 4). Female age (mean 36.1 vs. 35.7 years) and number of embryos transferred (mean 1.6 in each group) were comparable between groups. No statistically significant differences in clinical outcome were observed (PR: 67.5 % vs. 59.6 %, respectively; cPR: 59.2 % vs. 54.8 %, respectively; LBR: 53.3 % vs. 48.4 %, respectively, and MR: 5.9 % vs. 6.4 %, respectively) after treatment with hCG vs. medium.

**Table 4:** Clinical outcome in cohort B: hCG administration on day 5

	Transfer with top blastocysts			Transfer with non-top blastocysts		
Characteristics	hCG (n = 169)	culture medium (n = 188)	*P*-Value	hCG (n = 341)	culture medium (n = 306)	*P*-Value
Female age (years) mean *± s.d.* (range)	36.1 *± 4.0* (22–43)	35.7 *± 4.3* (20–43)	0.331	37.6 *± 3.9* (27–43)	37.3 *± 4.2* (25–43)	0.330
Endometrium height (mm) mean *± s.d.*	10.2 *± 1.5*	9.9 *± 1.6*	0.102	9.8 *± 1.7*	9.7 *± 1.6*	0.619
No. Embryos transferred Embryos / ET mean *± s.d*.	274 1.6 *± 0.5*	302 1.6 *± 0.5*	0.781	594 1.7 *± 0.5*	547 1.8 *± 0.4*	0.208
Proportion of difficult ETs	10.7 %	9.0 %	0.610	10.6 %	10.8 %	0.926
No. Pregnancies (PR)	114 (67.5 %)	112 (59.6 %)	0.123	147 (43.1 %)	149 (48.7 %)	0.155
No. Clinical pregnancies (cPR)	100 (59.2 %)	103 (54.8 %)	0.404	113 (33.1 %)	125 (40.8 %)	0.042
No. Implanted embryos (IR)	123 (44.9 %)	128 (42.4 %)	0.545	130 (21.9 %)	148 (27.1 %)	0.042
No. Births (LBR)	90 (53.3 %)	91 (48.4 %)	0.360	98 (28.7 %)	107 (35.0 %)	0.089
No. Babies born	108	112		110	126	
No. Miscarriages (MR)	10 (5.9 %)	12 (6.4 %)	0.855	15 (4.4 %)	18 (5.9 %)	0.392

Numbers or percentages respectively are given in brackets, s.d.: standard deviation

IVF characteristics and clinical outcome of cohort B. Miscarriage rate was defined as pregnancy loss after the detection of foetal heartbeat

Furthermore, 647 patients had a transfer with non-top blastocyst(s), whereas 341 received an intrauterine injection with hCG prior to transfer and 306 received an injection with medium. A mean number of 1.7 vs. 1.8 embryos was transferred. No statistically significant differences were observed in PR (43.1 % vs. 48.7 %, respectively), LBR (28.7 % vs. 35.0 %, respectively), and MR (4.4 % vs. 5.9 %, respectively) after injection with hCG vs. medium. However, cPR (33.1 % vs. 40.8 %, respectively) as well as IR (21.9 % vs. 27.1 %, respectively) were significantly higher in the controls (Table 4).

In cohort B, the twin birth rate after top blastocyst transfer was 10.0 % (hCG) vs. 11.0 % (control), and 6.1 % (hCG) vs. 8.4 % (control), respectively with the same number of embryos transferred in the groups (Table 2). No statistically significant difference between the groups was found.

#### Benefit of hCG application in relation to female age

On the same data-set we investigated a possible correlation between age and hCG administration. Two age-groups were defined: women <38 years (482 ETs) and ≥ 38 years (522 ETs). In the younger group, after treatment with hCG vs. medium, we found similar PR (60.4 % vs. 60.2 %, respectively) and LBR (47.1 % vs. 48.7 %, respectively). In women ≥ 38 years PR amounted to 41.4 % (hCG) vs. 44.1 % (control), and LBR 26.6 % (hCG) vs. 30.8 % (control), respectively. Quality of blastocysts transferred was similar in the groups and clinical outcome statistically not significant (data not shown).

## Discussion

In our study we investigated the impact of intrauterine hCG administration prior transfer in relation to blastocyst morphology in a large study population that includes birth outcome as primary outcome. In contrast to previous findings after cleavage stage embryo transfers, but in accordance with studies on blastocyst transfers, the present study did not show that intrauterine hCG application provides additional benefit in various settings [27]. In fact, neither intrauterine hCG injection on day three nor on day five had a beneficial effect on PR, DR, or IR. This was true in transfers with top blastocysts as well as in those with non-top blastocysts.

Several studies postulate that hCG is a key-player in the implantation process [8, 9, 14–18]. As one of the first signalling molecules, hCG is already secreted by cleavage stage embryos. In blastocysts, hCG is then exclusively produced by the trophectoderm (TE) cells. Human chorionic gonadotropin isoforms and the released amount of hCG were found to be related to the developmental stage, but also to the morphological quality of the blastocyst and to the TE grading in particular [10, 28–31]. In line with these findings, TE quality was suggested to be the most important predictor for implantation [32–34]. It has recently been assumed that decidual cells select against embryos lacking fitness including those with insufficient hCG production [35]. Thus, our study aimed to investigate a possible positive effect of intrauterine hCG injection on implantation and clinical outcome in blastocyst transfers with regard to blastocyst morphology.

Our finding, that hCG did not improve clinical outcome neither in top-blastocyst transfers nor in non-top blastocyst transfer is in contradiction to some previous papers reporting positive results from cleavage stage embryo transfers (Table 5). Mansour and co-workers found in a randomised trial including 212 cycles that application of 500 IU hCG prior to transfer of cleavage stage embryos significantly improves pregnancy rates [19]. In a similar approach, Zarei and colleagues reported an increase in PR by intrauterine injection of 250 μg recombinant hCG (equivalent to 6,500 IU) prior to transfer on day three [20]. In both studies, the age of patients was substantially lower compared with our cohorts (Table 5). Advanced female age is the most limiting factor for IVF success. As some of the studies that have found a beneficial effect of hCG administration were done on younger patients, we investigated a possible benefit of hCG administration on patients with advanced maternal age. However, no benefit of hCG administration was found for different age groups 20 to 38 years or 38 to 43 years.

**Table 5:** Overview of recent studies on intrauterine hCG infusion

Study	No. of ETs and patients	Maternal Age (Mean)	Type and Day of ET	hCG infusion/ hCG source	IR	cPR	LBR
Mansour et al., 2011 [19]	212 ETs in first IVF/ICSI cycle (male factor)	Control: 28.3a hCG: 28.4a	Fresh Day 2 or 3	500 IU purified urinary hCG (IBSA) 7 min prior ET	Control: 29.5 % hCG: 41.6 %	Control: 60 % hCG: 75 %	
Rebolloso et al., 2013 [22]	121 ETs IVF/ICSI cycles (38 in hCG group)	n.d.	n.d. Day 3 (n = 79) Day 5 (n = 42)	500 IU urinary hCG (n.d.) 7 min before ET	Control: 17.5 % hCG: 17.7 %	Control: 26.3 % hCG: 26.5 %	
Zarei et al., 2014 [20]	182 ETs in first IVF/ICSI cycle	Control: 31.3a hCG: 29.9a	Fresh Day 3	250 μg rhCG (Ovitrelle) (equivalent to 6,500 IU) 12 min prior ET	Control: 22.4 % hCG: 36.9 %	Control 18.4 % hCG: 32.1 %	
Santibañez et al., 2014 [21]	210 IVF and ICSI cycles including donor and autologous ETs	Control: 37.3a hCG: 36.4a	Fresh and frozen Day 3	500 IU purified urinary hCG (Choragon) 4 min prior ET		Control: 33.0 % hCG: 50.4 %	
Riboldi et al., 2013 [12]	99 ETs in women >35a, > 2 IVF attempts	Control: 39.2a hCG: 39.2a	Frozen Blastocysts Day (n.d.)	500 IU rhCG (n.d.) 6 hours prior ET		Control: 36.1 % hCG 39.3 %	
Hong et al., 2014 [27]	300 ETs with or without PGS	Control: 35.1a hCG: 35.0a	Fresh and frozen Day 6	500 IU urinary hCG (Novarel) 3 min prior ET	Control: 48.1 % hCG: 44.2 %	Control: 58.8 % hCG: 52.0 %	
Our results	182 ETs in women ≤43a without RIF	Control: 35.5a hCG: 36.1a All: 35.8a	Fresh Day 5	500 IU urinary hCG (Pregnyl) 2 days prior ET	*Top Blastocysts* Control: 42.1 % hCG: 40.0 % *Non-top Blastocysts* Control 15.6 % hCG 15.9 %	*Top Blastocysts* Control: 57.1 % hCG: 52.1 % *Non-top Blastocysts* Control: 20.5 % hCG: 19.5 %	*Top Blastocysts* Control: 53.1 % hCG: 50.0 % *Non-top Blastocysts* Control: 18.2 % hCG: 17.1 %
Our results	1004 ETs in women ≤43a without RIF	Control: 36.7a hCG: 37.1a All: 36.9a	Fresh Day 5	500 IU urinary hCG (Pregnyl) 3 min prior ET	*Top Blastocysts* Control: 42.4 % hCG: 44.9 % *Non-top Blastocysts* Control: 27.1 % hCG: 21.9 %	*Top Blastocysts* Control: 54.8 % hCG: 59.2 % *Non-top Blastocysts* Control: 40.8 % hCG: 33.1 %	*Top Blastocysts* Control: 48.4 % hCG 53.3 % *Non-top Blastocysts* Control: 35.0 % hCG: 28.7 %

a: years; cPR: clinical pregnancy rate; IR: implantation rate; LBR: live birth rate; n.d.: not defined; RIF: recurrent implantation failure

Summary of different studies analyzing the putative benefit of intrauterine hCG supplementation in correlation to the IVF outcome (IR, cPR and LBR)

In line with our previous suggestion, that a beneficial effect of hCG application is dependent on the stage of the embryo(s) transferred, a recent study investigated blastocyst transfers in women >35 years [12]. No increase in cPR was observed in the hCG group. However, authors reported after further sub-grouping according to blastocyst quality, a higher PR with hCG injection when poor quality blastocysts were transferred [12]. This finding could not be observed in our study, which could be explained by the fact that in the cited study, the number of patients per group was substantially low. Our observations suggest that a putative decreased production of implantation supporting mediators in embryos with poor morphology cannot be overcome by intrauterine administration of hCG. On the contrary, a statistically significant negative effect of hCG administration prior to transfer on cPR and IR was observed in the poor morphology group. This might support the hypothesis that a well coordinated signalling is most crucial for a proper implantation and the enhancing of one signal might even worsen the situation. Thus, more trials addressing this issue might be needed. However, LBR was not statistically lower with hCG application (Table 4).

Moreover, in line with our results, the study of Rebolloso and colleagues did not observe any improvement in clinical outcome after administration of hCG [22]. This randomised study performed on a small cohort, including 38 patients who received 500 IU hCG and 83 patients in the control group. Transfers included both cleavage stage and blastocyst transfers. Interestingly, the authors reported a statistically significantly higher twin pregnancy rate after hCG injection (66 % vs. 8.3 % in hCG vs. control). This finding could not be confirmed in our data set. No differences in the two study cohorts were observed.

Consistent with our study, a recently published paper reported no impact of intrauterine hCG injection prior to blastocyst transfer [27]. This investigation included 300 fresh or frozen transfers. No statistically significant differences in IR or cPR between the hCG and control groups were observed. This was also true for a subgroup of patients receiving a transfer after comprehensive chromosome screening. These findings confirm our results, however, the cited study was discontinued before completion of the calculated sample size. Most important, the quality of blastocysts transferred was not further investigated, although it is well accepted that besides female age and the cause of infertility, blastocyst morphology is one of the most important parameters that determines whether or not implantation is successful [25, 36]. Data analysis in respect to the quality of transferred blastocysts is crucial, as embryo quality is probably an indicator of both: the hCG production and the probability of implantation [10, 11]. Our study clearly demonstrates that neither an intrauterine injection of hCG two days prior to ET nor an injection on the day of blastocyst transfer can overcome the reported reduced implantation chances of a poor quality blastocyst. This suggestion is in line with previous publication of Eftekhar *et al*. [37] and Ben-Meir *et al*. [38]. Both research groups observed that the subcutaneous administration of recombinant hCG in frozen-thawed ET cycles on the day of transfer does not have any advantages in terms of pregnancy and implantation rates. Further, in line with several previous reports, we did not observe an increase in miscarriages in the hCG groups when compared to controls. These results confirm previous findings [19–22, 27]. In conclusion, this large prospective randomised study could not find any beneficial effect of intrauterine hCG injection (500 IU) in blastocyst transfers. This was true for top as well as for non-top blastocyst. The strengths of the present study are that it is an RCT with a high number of analysed subjects in two different set-ups, a unique stimulation scheme and the evaluation of the outcome in correlation to embryo quality. However, it has to be mentioned that the patient cohort receiving hCG injection on day 3 in our study was substantially smaller as compared to cohort B receiving hCG on day 5 prior to transfer and therefore this group did not reach the calculated power analysis. The main limitation is the restriction to fresh blastocyst transfers. The WOI is assumed to be altered by high dosages of exogenous hCG (as it is done during cycles of COH) [39]. Thus, a beneficial effect of intrauterine hCG administration in cryo-cycles cannot be excluded as the transfer of vitrified/thawed blastocysts is often suggested to be more physiologic. Furthermore, our results apply only to the patients clientele investigated in this study (autologous IVF cycles in patients ≤ 43 years). Additionally, as source and brand were found to be crucial for the mode of hCG action [18], we cannot exclude that other hCG concentrations or other hCG preparations might have different impacts.

## Conclusions

The high number of IVF cycles with failed implantation despite the transfer of apparently good and viable embryos with respect to their morphology has led to the adoption of adjuvant therapies with the aim to improve endometrial receptivity and implantation rates. However, one must keep in mind that the molecular background of the implantation process remains a complex issue, and intervention therapies are not as simple as has often been suggested. Further studies are crucial for a better understanding of the molecular mechanisms of embryonic-maternal cross-talk, taking into consideration the possible differences in hCG molecules such as embryonic vs. endometrial vs. recombinant hCG, and to implement truly effective and innovative therapies in assisted reproduction.

## Abbreviations

BMI: Body mass index
cPR: Clinical pregnancy rates
ET: Embryo transfer
FSH: Follicle-stimulating hormone
GnRHa: Gonadotrophin‐releasing hormone agonist
hCG: Human chorionic gonadotropin
IMSI: Intracytoplasmic morphologically selected sperm injection
IR: Implantation rate
IVF: In *vitro* fertilization
LBR: Live birth rate
MII: Metaphase II
MR: Miscarriage rates
n.d.: Not defined
n.s.: Not significant
OPU: Ovum pick-up
PGS: Pre-implantation genetic screening
PR: Pregnancy rate
rhCG: Recombinant hCG
uNK: Uterine natural killer
s.d.: Standard deviation
SPSS: Statistical package for social sciences
TE: Trophectoderm
WOI: Window of implantation

## References

[1] Koot YE, Boomsma CM, Eijkemans MJ, Lentjes EG, Macklon NS (2011). Recurrent pre-clinical pregnancy loss is unlikely to be a ‘cause’ of unexplained infertility. Hum Reprod.

[2] Ord T (2008). The scourge: moral implications of natural embryo loss. Am J Bioeth.

[3] Fluhr H, Bischof-Islami D, Krenzer S, Licht P, Bischof P, Zygmunt M (2008). Human chorionic gonadotropin stimulates matrix metalloproteinases-2 and −9 in cytotrophoblastic cells and decreases tissue inhibitor of metalloproteinases-1, −2, and −3 in decidualized endometrial stromal cells. Fertil Steril.

[4] Cole LA (2010). Biological functions of hCG and hCG-related molecules. Reprod Biol Endocrinol.

[5] Tsampalas M, Gridelet V, Berndt S, Foidart JM, Geenen V, Perrier d’Hauterive S (2010). Human chorionic gonadotropin: a hormone with immunological and angiogenic properties. J Reprod Immunol.

[6] Tapia-Pizarro A, Argandoña F, Palomino WA, Devoto L (2013). Human chorionic gonadotropin (hCG) modulation of TIMP1 secretion by human endometrial stromal cells facilitates extravillous trophoblast invasion in vitro. Hum Reprod.

[7] Bourdiec A, Calvo E, Rao CV, Akoum A (2013). Transcriptome analysis reveals new insights into the modulation of endometrial stromal cell receptive phenotype by embryo-derived signals interleukin-1 and human chorionic gonadotropin: possible involvement in early embryo implantation. PLoS One.

[8] Edwards RG (2007). Chorionic gonadotrophin, genes and embryonic signals regulating the implantation window. Reprod Biomed Online.

[9] Bonduelle ML, Dodd R, Liebaers I, Van Steirteghem A, Williamson R, Akhurst R (1988). Chorionic gonadotrophin-beta mRNA, a trophoblast marker, is expressed in human 8-cell embryos derived from tripronucleate zygotes. Hum Reprod.

[10] Dokras A, Sargent IL, Barlow DH (1993). Human blastocyst grading: an indicator of developmental potential?. Hum Reprod.

[11] Brosens JJ, Salker MS, Teklenburg G, Nautiyal J, Salter S, Lucas ES (2014). Uterine selection of human embryos at implantation. Sci Rep.

[12] Riboldi M, Barros B, Piccolomini M, Alegretti JR, Motta ELA, Serafini PC (2013). Does the intrauterine administration of rhCG before vitrified blastocysts transfer improves the potential of pregnancies when using blastocysts of inferior morphological grading?. Fertil Steril.

[13] Licht P, Fluhr H, Neuwinger J, Wallwiener D, Wildt L (2007). Is human chorionic gonadotropin directly involved in the regulation of human implantation?. Mol Cell Endocrinol.

[14] Licht P, Russu V, Wildt L (2001). On the role of human chorionic gonadotropin (hCG) in the embryo-endometrial microenvironment: implications for differentiation and implantation. Semin Reprod Med.

[15] Sálker M, Teklenburg G, Molokhia M, Lavery S, Trew G, Aojanepong T (2010). Natural selection of human embryos: impaired decidualization of endometrium disables embryo-maternal interactions and causes recurrent pregnancy loss. PLoS One.

[16] Paiva P, Hannan NJ, Hincks C, Meehan KL, Pruysers E, Dimitriadis E (2011). Human chorionic gonadotrophin regulates FGF2 and other cytokines produced by human endometrial epithelial cells, providing a mechanism for enhancing endometrial receptivity. Hum Reprod.

[17] Srivastava A, Sengupta J, Kriplani A, Roy KK, Ghosh D (2013). Profiles of cytokines secreted by isolated human endometrial cells under the influence of chorionic gonadotropin during the window of embryo implantation. Reprod Biol Endocrinol.

[18] Racicot KE, Wünsche V, Auerbach B, Aldo P, Silasi M, Mor G (2014). Human Chorionic Gonadotropin Enhances Trophoblast-Epithelial Interaction in an In Vitro Model of Human Implantation. Reprod Sci.

[19] Mansour R, Tawab N, Kamal O, El-Faissal Y, Serour A, Aboulghar M (2011). Intrauterine injection of human chorionic gonadotropin before embryo transfer significantly improves the implantation and pregnancy rates in in vitro fertilization/intracytoplasmic sperm injection: a prospective randomized study. Fertil Steril.

[20] Zarei A, Parsanezhad ME, Younesi M, Alborzi S, Zolghadri J, Samsami A (2014). Intrauterine administration of recombinant human chorionic gonadotropin before embryo transfer on outcome of in vitro fertilization/ intracytoplasmic sperm injection: A randomized clinical trial. Iran J Reprod Med.

[21] Santibañez A, García J, Pashkova O, Colín O, Castellanos G, Sánchez AP (2014). Effect of intrauterine injection of human chorionic gonadotropin before embryo transfer on clinical pregnancy rates from in vitro fertilisation cycles: a prospective study. Reprod Biol Endocrinol.

[22] Rebolloso MM, Rosales De Leon JC, Galache Vega P, Santos-Haliscak R, Diaz-Spindola P, Gonzalez Vega O (2013). Do intrauterine injection of human chorionic gonadtropin (hcg) before embryo transfer increases implantation and pregnancy rates in patients undergoing in vitro fertilization?. Fertil Steril.

[23] Zech NH, Lejeune B, Stecher A, Puissant F, Vanderzwalmen S, Zech H (2007). A prospective evaluation on the optimal time for selecting a single embryo for transfer: day 3 versus day 5. Fertil Steril.

[24] Vanderzwalmen P, Hiemer A, Rubner P, Bach M, Neyer A, Stecher A (2008). Blastocyst development after intracytoplasmic morphologically selected sperm injection (IMSI) is directly correlated with the morphological integrity of human sperm nuclei. Reprod Biomed Online.

[25] Gardner DK, Lane M, Stevens J, Schlenker T, Schoolcraft WB (2000). Blastocyst score affects implantation and pregnancy outcome: towards a single blastocyst transfer. Fertil Steril.

[26] Spitzer D, Haidbauer R, Corn C, Stadler J, Wirleitner B, Zech NH (2012). Effects of embryo transfer quality on pregnancy and live birth delivery rates. J Assist Reprod Genet.

[27] Hong KH, Forman EJ, Werner MD, Upham KM, Gumeny CL, Winslow AD (2014). Endometrial infusion of human chorionic gonadotropin at the time of blastocyst embryo transfer does not impact clinical outcomes: a randomized double-blind, placebo-controlled trial. Fertil Steril.

[28] Woodward BJ, Lenton EA, Turner K (1993). Human chorionic gonadotrophin: embryonic secretion is a time-dependent phenomenon. Hum Reprod.

[29] Butler SA, Luttoo J, Freire MO, Abban TK, Borrelli PT, Iles RK (2013). Human chorionic gonadotropin (hCG) in the secretome of cultured embryos: hyperglycosylated hCG and hCG-free beta subunit are potential markers for infertility management and treatment. Reprod Sci.

[30] Xiao-Yan C, Jie L, Dang J, Tao L, Xin-Ru L, Guang-Lun Z (2013). A highly sensitive electrochemiluminescence immunoassay for detecting human embryonic human chorionic gonadotropin in spent embryo culture media during IVF-ET cycle. J Assist Reprod Genet.

[31] Wang H, Zhang R, Han D, Liu C, Cai J, Bi Y (2014). Association of human chorionic gonadotropin level in embryo culture media with early embryo development. Nan Fang Yi Ke Da Xue Xue Bao.

[32] Thompson SM, Onwubalili N, Brown K, Jindal SK, McGovern PG (2013). Blastocyst expansion score and trophectoderm morphology strongly predict successful clinical pregnancy and live birth following elective single embryo blastocyst transfer (eSET): a national study. J Assist Reprod Genet.

[33] Ahlström A, Westin C, Reismer E, Wikland M, Hardarson T (2011). Trophectoderm morphology: an important parameter for predicting live birth after single blastocyst transfer. Hum Reprod.

[34] Desai J, Holt-Shore V, Torry RJ, Caudle MR, Torry DS (1999). Signal transduction and biological function of placenta growth factor in primary human trophoblast. Biol Reprod.

[35] Macklon NS, Brosens JJ (2014). The Endometrium as a Sensor of Embryo Quality. Biol Reprod.

[36] Van den Abbeel E, Balaban B, Ziebe S, Lundin K, Cuesta MJ, Klein BM (2013). Association between blastocyst morphology and outcome of single-blastocyst transfer. Reprod Biomed Online.

[37] Eftekhar M, Rahmani E, Eftekhar T (2012). Effect of adding human chorionic gonadotropin to the endometrial preparation protocol in frozen embryo transfer cycles. Int J Fertil Steril.

[38] Ben-Meir A, Aboo-Dia M, Revel A, Eizenman E, Laufer N, Simon A (2010). The benefit of human chorionic gonadotropin supplementation throughout the secretory phase of frozen-thawed embryo transfer cycles. Fertil Steril.

[39] Bernardini L, Moretti-Rojas I, Brush M, Rojas FJ, Balmaceda JP (2013). Failure of hCG/LH receptors to stimulate the transmembrane effector adenylyl cyclase in human endometrium. Adv Biosci Biotechnol.

